# The impact of modified incision height and surgical procedure on trichiasis surgery outcomes: Results of the maximizing trichiasis surgery success (MTSS) randomized trial

**DOI:** 10.1371/journal.pntd.0012034

**Published:** 2024-09-03

**Authors:** Emily W. Gower, Alemayehu Sisay, Belay Bayissasse, Dawit Seyum, Jerusha Weaver, Beatriz Munoz, Alexander P. Keil, Andrea Bankoski, Kristin M. Sullivan, Hashiya Kana, Fisseha Admassu, Demissie Tadesse, Shannath L. Merbs

**Affiliations:** 1 Department of Epidemiology, University of North Carolina at Chapel Hill, Chapel Hill, North Carolina, United States of America; 2 Orbis International Ethiopia, Addis Ababa, Ethiopia; 3 Department of Ophthalmology, Johns Hopkins School of Medicine, Baltimore, Maryland, United States of America; 4 Department of Ophthalmology, University of Gondar, Gondar, Ethiopia; 5 CBM International, Addis Ababa, Ethiopia; 6 Department of Ophthalmology and Visual Sciences, University of Maryland School of Medicine, Baltimore, Maryland, United States of America; RTI International, UNITED REPUBLIC OF TANZANIA

## Abstract

**Background:**

Poor surgical outcomes remain a problem in trachoma-endemic countries working to reach elimination thresholds. Methods to improve outcomes could positively impact programmatic success.

**Methods:**

This parallel, three-armed clinical trial conducted in Ethiopia randomized individuals with previously unoperated trachomatous trichiasis (TT) to receive surgery utilizing one of three approaches: bilamellar tarsal rotation with a 3 mm incision height (BLTR-3), BLTR with 5 mm incision height (BLTR-5) and posterior lamellar tarsal rotation (PLTR). We followed participants for one year. The primary outcome was post-operative trichiasis (PTT). Secondary outcomes were eyelid contour abnormalities (ECA) and pyogenic granulomata.

**Findings:**

We randomized and operated on 4,914 individuals with previously unoperated TT (6,940 eyes). Primary analyses include 6,815 eyes with follow-up. Overall, 1,149 (16.9%) eyes developed PTT. The risk difference for PTT was minimal comparing BLTR-3 and PLTR (adjusted risk difference [aRD] 1.8% (98.3%CI: -0.5–4.2%)), but significantly higher for BLTR-5 surgeries compared to BLTR-3 (aRD: 6.7% (3.9–9.4%)) and PLTR (aRD: 8.6% (5.9–11.3%)). BLTR-5 had the lowest ECA (6.1% versus 9.6% BLTR-3, 11.2% PLTR) and granuloma rates (5.2% versus 6.5% BLTR-3 and 7.5% PLTR). One eyelid operated with PLTR experienced an eyelid margin division; four BLTR-3 and eight BLTR-5 eyelids experienced excessive bleeding.

**Interpretation:**

We do not recommend modifying the BLTR incision height of 3 mm. Overall, we did not find a significant difference in PTT between BLTR-3 and PLTR in terms of PTT or ECA.

**Trial registration:**

Registration number: NCT03100747; ClinicalTrials.gov

Full study protocol available at (https://doi.org/10.15139/S3/QHZXWD)

## Author summary

Trachomatous trichiasis (TT) surgery is the primary way to manage in-turned eyelashes in trachoma-endemic settings. The goal of this study was to understand what procedure has the best outcomes. We compared three ways of doing TT surgery: bilamellar tarsal rotation (BLTR) surgery with the World Health Organization recommended 3 mm incision height, BLTR with the incision height increased to 5 mm and posterior lamellar tarsal rotation (PLTR). We hypothesized that increasing the BLTR incision height to 5 mm would reduce post-operative trichiasis (PTT) compared with the 3 mm height. On the contrary, this study demonstrates that increasing the incision height of BLTR surgeries does not improve PTT rates. It also suggests that BLTR and PLTR surgeries can have similar outcomes in some settings. Further research is needed to examine the impact of training order on surgery outcomes. The study also investigated whether surgeon performance on a particular procedure depends on the order they were trained. The current study does not suggest that converting BLTR to PLTR surgeons reduces PTT rates. However, given the small number of conversion surgeons in this study, further research is needed to evaluate whether retraining BLTR surgeons is warranted.

## Introduction

Approximately 1.7 million cases of trachomatous trichiasis (TT) globally currently need management.[1] Surgery is the preferred management strategy, and surgery to correct TT in trachoma-endemic countries typically is performed with one of two surgical procedures, bilamellar tarsal rotation (BLTR) or posterior lamellar tarsal rotation (PLTR), which is a modification of the Trabut procedure [2]. Each procedure involves making an incision across the width of the eyelid, followed by placing sutures to externally rotate the eyelid margin and trichiatic eyelashes back to their normal anatomic position. BLTR utilizes a full-thickness incision, and the World Health Organization (WHO) recommends placing the incision 3 millimeters (mm) above the eyelid margin. PLTR involves a partial-thickness incision of the eyelid through the tarsal conjunctiva and tarsus, 3 mm from the eyelid margin. The actual distance from the eyelid margin to the incision, known as the incision height, may vary unintentionally from the targeted 3 mm incision height [3]. Incision height variation is probably more common with BLTR given the differences between the way the procedures are performed.

Over the past 10 years, WHO TT surgery guidelines have changed as new data have become available. A randomized trial conducted in Ethiopia showed that the one-year PTT risk was significantly lower after PLTR compared to BLTR with a standard 3 mm incision height [4]. The results led to the current WHO recommendation that new surgeons be taught PLTR, while experienced surgeons continue with the procedure they typically use (either BLTR or PLTR) [5]. The study used PLTR surgeons who were retrained on BLTR for the trial, and no data are available to determine whether prior experience with PLTR introduced an unintended bias in the study. Evidence from a randomized clinical trial in Tanzania suggested that eyelids with a central BLTR incision scar height of <4.5 mm were 2.5-fold more likely to have post-operative TT (PTT) than eyelids with a higher incision scar height [3]. Thus, we designed the Maximizing Trichiasis Surgery Success (MTSS) trial to determine whether increasing the BLTR incision height from 3 to 5 mm modified the one-year PTT risk and to compare the risk of PTT between PLTR and each of the BLTR incision height groups, separately. We also examined the impact of the procedure on eyelid contour abnormalities (ECA) and pyogenic granuloma formation. Finally, we assessed whether the order surgeons learned each procedure impacted surgical outcomes.

## Methods

Full details of the trial methods have been published elsewhere [6]. We describe key methods below.

### Ethics statement

This trial is registered at ClinicalTrials.gov (Identifier: NCT03100747). The National Research Ethics Review Committee of Ethiopia, the Food Medicine Healthcare and Control Authority of Ethiopia, and the Institutional Review Boards at the University of North Carolina at Chapel Hill and University of Maryland School of Medicine approved this study. A Data and Safety Monitoring Committee (DSMC) was convened with four voting members and a non-voting member from the funding organization, the US National Eye Institute. The DSMC met biannually. The Ethiopian oversight committees also monitored the trial through annual reports and periodic field visits.

### Study design and population

In this randomized trial, we allocated participants on a 1:1:1 basis to BLTR with a 3 mm incision height (BLTR-3), BLTR with a 5 mm incision height (BLTR-5), or PLTR. Participants with both eyes needing TT surgery received the same surgery type for both eyelids. We enrolled participants in the Hadiya, Gedio, and Keffa zones and the Yem, Amaro, and Burji special woredas of Southern Nations Nationalities and Peoples Regional State of Ethiopia. We identified potential participants through two screening approaches: community mobilization with centralized screening by an integrated eye care worker and house-to-house screening by community health assistants who then brought potential trichiasis cases to a central location for final assessment by an integrated eye care worker [7].

Individuals were eligible for the trial if they met the following criteria: at least one upper eyelid with previously unoperated TT, aged ≥18 years, willingness to comply with all trial procedures and be randomized to any of the surgical procedures, and no plans to move from the region during the trial. We excluded individuals who were unable to provide informed consent or if all otherwise eligible eyes were phthisical. Individuals who were interested in participating in the trial provided written informed consent on the day of surgery. A certified consent specialist met individually with each person to review the consent form and ask if they had any questions prior to signing.

Two types of surgeons were utilized for this trial: those newly trained simultaneously on BLTR and PLTR procedures (“dually-trained surgeons”) and those originally trained on BLTR, but later trained on PLTR (“conversion surgeons”). Conversion surgeons were required to perform ≥100 PLTR surgeries before performing trial surgeries.

### Intervention

BLTR-3 and PLTR surgeries were performed as described in the WHO’s *Trichiasis Surgery for Trachoma* manual [2]. Surgeons performed BLTR surgery with a TT clamp [8]. When BLTR-5 was assigned, surgeons followed the standard BLTR procedure but increased the incision height to 5 mm above the eyelid margin. For BLTR surgeries, surgeons used a caliper and pen to mark the desired incision location on the skin and then made the full-thickness incision along this mark. PLTR incisions were made without measuring or marking to replicate the standard PLTR procedure.

#### Follow-up visits

We followed participants 1 day, 2 weeks, 6 weeks and 12–18 months after surgery. At each visit, trained examiners assessed both eyelids for the presence of trichiatic eyelashes (number and location), evidence of epilation, ECA, and pyogenic granuloma. Photographs were taken of each eyelid, first with the smartphone camera below the eye, angled up, with the participant looking up. Then with the participant looking forward at the camera. Next images were taken with the camera off-set 45 degrees from center to assess whether in-turned eyelashes touched the globe and to obtain an alternate view of the eyelid thickness and contour.

### Outcomes

Trained integrated eye care workers not involved with the surgery conducted all follow-up visits. These examiners assessed participants for the presence of three outcomes (PTT, ECA, and granuloma) and took eye-level photographs at each visit. Each examiner was required to achieve a minimum kappa of 0.7 against a gold standard grader (EWG, SLM) for each outcome prior to certification for this study.

#### Primary outcome

The primary outcome was cumulative PTT incidence, defined as PTT noted at the 6-week or 12-18-month visits. We considered PTT to be present if at least one eyelash was touching the globe or there was clear evidence of epilation. A photograph grader (HK), masked to treatment assignment, evaluated each eyelid for PTT using the front-facing image with the participant looking up to allow visualization of eyelash-globe contact. When an eyelid had a questionable trichiatic eyelash, we also examined photographs taken in primary gaze and taken at a 45-degree angle from the side, with the participant in primary gaze. Field and photograph grades were compared. Occasionally, photo-grading indicated PTT when field grading did not. In these instances, our senior ophthalmologist (SLM) reviewed the photographs in a masked fashion and provided a final grade. If PTT was clearly visible to her, we utilized the photograph grade as the primary grade. When the field indicated PTT was present, but the photograph grader did not, we maintained the field grade. We classified TT severity using our standard grading system, which considers the number of eyelashes touching the globe and the proportion of the eyelid epilated [9].

#### Secondary outcomes

We assessed two secondary outcomes, ECA and pyogenic granuloma. We classified ECAs based on vertical deviation of the eyelid margin from the natural contour and proportion of the eyelid length affected [12]. Because ECA severity may change over time, we utilized the ECA assessment from the participant’s last study visit. We also photograded all study eyelids for ECA. When the field and photograph disagreed, a senior grader (EWG, SLM) determined the final photograph grade. Primary ECA analyses utilize the photograph grade. Sensitivity analyses use field grades. We defined pyogenic granuloma as a sessile growth of at least 2 mm on the upper tarsal conjunctiva. The examiner looked for the presence of granuloma at each follow up visit. When present, they removed it and took images of the eyelid before and after granuloma removal.

### Sample size

We designed this trial to have 80% power to detect a 25% relative difference for each comparison; a two-sided alpha level was set to 0.0167 to account for the three comparisons. We determined that we would need a sample size of 6,920 eyelids assuming a 15% PTT incidence in the BLTR-3 group and 5% loss to follow-up [6]. Based on prior work, we assumed 40% of participants would have bilateral TT and assumed an intra-class correlation coefficient of 0.24. Given these assumptions, we estimated 5,001 participants were needed to meet the eyelid sample size target.

### Randomization and masking

Staff at the University of North Carolina used a custom randomization program to assign participants to their treatment group using permuted block sizes of 6 and 12. Treatment arm assignments were preprinted on the surgical evaluation data collection form, which was sealed in an opaque envelope with the associated participant identification number on the outside of the envelope. The treatment assignment envelope was opened by the surgical assistant only after the participant was in the operating room to prevent surgeons from selecting their next surgery based on the available assignments. Participants, baseline examiners, and outcome examiners were masked to treatment assignments. Given the differences in surgical scars for the BLTR and PLTR procedures, it was impossible to fully mask the field outcome examiners. Hence, we utilized photograph grading to confirm field grades for our primary outcomes, as the surgical skin scar was not visible at the angle the photographs were taken.

### Statistical methods

The pre-specified primary analyses comprised three pairwise comparisons of cumulative PTT incidence. We used logistic regression models with the generalized estimating equations approach to account for the correlation between two eyes of the same person. When participants had bilateral TT, the same surgeon performed the surgery on both eyes. We assumed that the minimal loss-to-follow-up has a negligible impact on the effect estimate and performed analyses among study participants with complete outcomes data. For each analysis, statistical significance for comparing differences between study arms is determined based on an alpha level of 0.0167 to account for the multiple testing that results from using pairwise comparisons. We conducted secondary analyses stratified by surgeon type (dually-trained or conversion) and adjusted for baseline TT severity. Data management and analyses were conducted using SAS version 9.4 (Cary, NC, USA).

### Role of the funding source

The funder had no role in data collection, analysis, or interpretation of this trial. The corresponding author had full access to the data and assumed final responsibility for the decision to submit the manuscript for publication.

## Results

**Fig 1:**
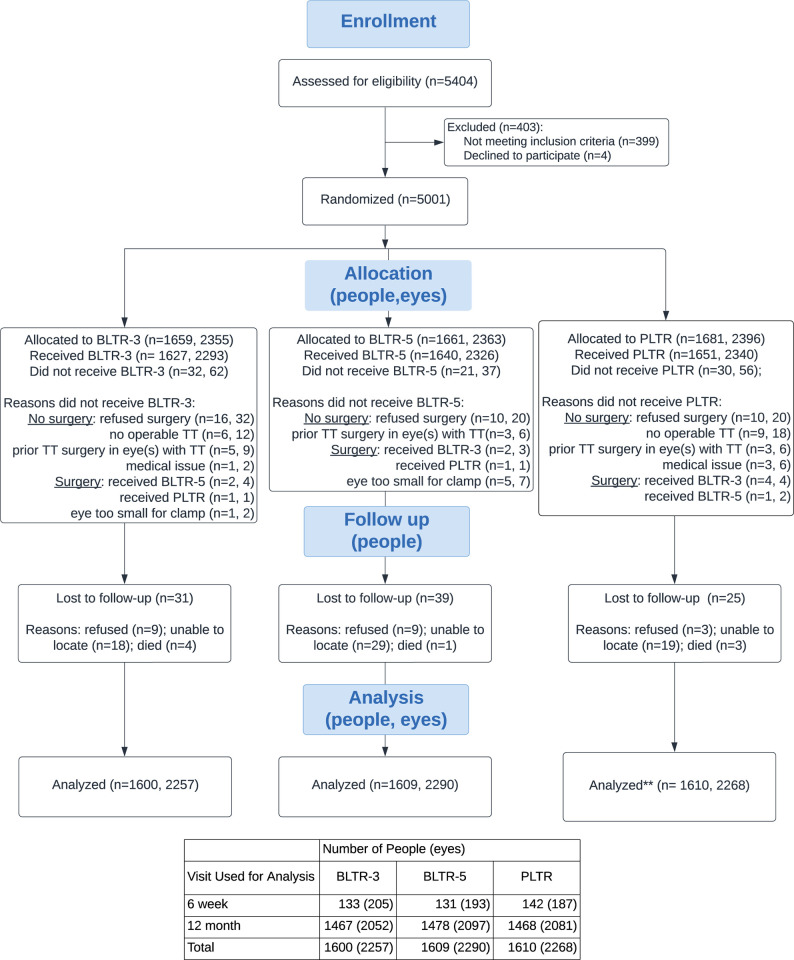
Participant Flow in a Study of Trachomatous Trichiasis Surgery Procedures. Notes: PLTR: posterior lamellar tarsal rotation; BLTR: bilamellar tarsal rotation; *21 people, 24 eyes enrolled out of sequence excluded from primary analyses.

Between April 2017 and April 2019, we screened 5,404 individuals with upper eyelid trichiasis for study enrollment. Of these, 5,001 individuals enrolled in the MTSS trial (**Fig 1**). Four individuals refused surgery. The remaining 399 individuals were not eligible, primarily due to having had prior TT surgery for TT-affected eyelids (n = 231; 57.8%). Among the 5,001 participants enrolled and randomized, 66 did not receive surgery as part of the study. The majority of these (n = 36) declined surgery after enrollment; upon further examination by a surgeon the remainder were deemed to have PTT only (n = 11), mild peripheral trichiasis without entropion (n = 15) or a medical contraindication for surgery that day (n = 4). Finally, due to an administrative error, 21 individuals enrolled on one day were assigned participant identification (id) numbers out of sequence. Although they received surgery with their assigned procedure, we excluded them from primary analyses. This error was caught the day it occurred, and the range of ids including these 21 was removed from the randomization sequence. New randomization envelopes with unique id numbers were added to the end of the series to account for removing this range. Ultimately, 4,914 individuals (6,940 eyes) received study-related surgery across the three study groups, performed by one of seven surgeons. Baseline characteristics were well balanced across the three study groups (**Table 1**). Seventy-five percent were women, and 41% had bilateral first-time TT surgery.

**Table 1:** Baseline Characteristics of Study Participants and Enrolled Eyes.

	Treatment Arm, N (column %)		
	BLTR-3	BLTR-5	PLTR
**Participant-level Characteristics**			
Total participants	1,631	1,648	1,635
**Age (years):**			
<40	291 (17.8)	314 (19.1)	311 (19.0)
40–60	1075 (65.9)	1041 (63.2)	1053 (64.4)
>60	265 (16.2)	293 (17.8)	271 (16.6)
% Female	1222 (74.9)	1261 (76.5)	1223 (74.8)
**Distance visual acuity worse than 20/400**			
One eye	268 (16.7)	270 (16.6)	271 (16.7)
Both eyes	226 (14.1)	226 (13.9)	221 (13.7)
**Both eyes in study**	668 (41.0)	689 (41.8)	669 (40.9)
**Eye-level Characteristics of Study Eyes†**			
**Total study eyes**	2299	2337	2304
Right eye	1138 (49.5)	1170 (50.1)	1140 (49.5)
**Number of eyelashes touching globe**			
None (epilating only)	164 (7.1)	157 (6.7)	173 (7.5)
1–4	1314 (57.2)	1414 (60.5)	1313 (57.0)
5–9	502 (21.8)	481 (20.6)	507 (22.0)
10+	318 (13.8)	285 (12.2)	311 (13.5)
**Epilation**			
None	1615 (70.2)	1678 (71.8)	1670 (72.5)
<1/3 of eyelid	156 (6.8)	137 (5.9)	150 (6.5)
1/3-2/3 of eyelid	91 (4.0)	101 (4.3)	102 (4.4)
>2/3 of eyelid	432 (18.8)	421 (18.0)	381 (16.5)
**Trichiasis Location(s)** *			
Nasal	1182 (51.4)	1148 (49.1)	1133 (49.2)
Central	1920 (83.5)	1979 (84.7)	1888 (81.9)
Temporal	1252 (54.5)	1240 (53.1)	1236 (53.6)
**Trichiasis severity**			
Mild	1028 (44.7)	1143 (48.9)	1072 (46.5)
Moderate	431 (18.7)	390 (16.7)	443 (19.2)
Severe	836 (36.4)	803 (34.4)	788 (34.2)
**Conjunctivalization of the eyelid margin** ‡			
None	27 (1.2)	28 (1.2)	18 (0.8)
Mild: 1–49% of eyelid length	132 (5.7)	149 (6.4)	141 (6.1)
Moderate/severe: ≥50% of eyelid length	2106 (91.6)	2135 (91.4)	2122 (92.1)
**Corneal scar grade**			
C0, C1: None or outside pupil margin	2044 (88.9)	2113 (90.4)	2047 (88.8)
C2a, C2b: obscures pupil margin	126 (5.5)	105 (4.5)	133 (5.8)
C2c, C2d: obscures pupil margin and central pupil	109 (4.7)	101 (4.3)	100 (4.3)
C3: covers cornea	13 (0.6)	17 (0.7)	23 (1.0)

Abbreviations: BLTR: Bilamellar tarsal rotation; PLTR: Posterior lamellar tarsal rotation.

*Locations are based on both trichiatic eyelashes and epilation, and locations are not mutually exclusive.

**†**Missing data: Distance visual acuity, 69 participants, 109 eyes; corneal scar, 9 eyes; conjunctivalization of eyelid margin, 82 eyes; Epilation, 6 eyes; Number of eyelashes touching globe, 1 eye; TT severity, 6 eyes; TT locations, 2 eyes.

‡Proportion of eyelid length where mucocutaneous junction is in front of meibomian gland openings

Over half of enrolled eyes had moderate or severe TT at baseline. The majority had trichiatic eyelashes emanating centrally, and 92% had at least moderate conjunctivalization of the eyelid margin (the mucocutaneous junction between the Meibomian gland openings and the eyelash bases for most of the eyelid margin width). Women were more likely to have severe TT than men, after adjusting for age (38% vs. 26%; chi-square p <0.001).

Follow-up rates were high, with 95% follow-up at 6 weeks and 91% at 12–18 months. 95 individuals (125 eyes) were not seen at either the 6-week or 12-18-month visit and were excluded from the analyses. Additionally, 24 eyelids received the wrong study procedure. Of these, nine eyelids assigned to BLTR received PLTR because the eyelid was too scarred to allow TT clamp placement. The remaining 15 eyes received the wrong procedure due to surgeon error. One eye assigned to BLTR-3 and one eye assigned to BLTR-5 received PLTR. Four eyes assigned to BLTR-3 received BLTR-5 and three eyes assigned to BLTR-5 received BLTR-3. Four eyes assigned to receive PLTR received BLTR-3, and two received BLTR-5. These eyes remained in their assigned treatment group for all analyses presented here (i.e., intention-to-treat), with the exception of one sensitivity analysis.

### Post-operative Trichiasis

By 12–18 months, 1,149 eyelids (16.9%) developed PTT; 996 were identified in the field, and an additional 153 were identified through photograph grading. Most PTT cases (82.0%) occurred between the 6-week visit and the 12-18-month visit, and the cumulative incidence of PTT ranged from 13.1% in the PLTR group to 22.0% in the BLTR-5 group (**Table 2**).

**Table 2:** Characteristics of Study Eyes at Last Follow up.

	Treatment Arm [N(%)]		
	BLTR-3	BLTR-5	PLTR
Total study eyes with follow up	2,257	2,290	2,268
**Post-operative trichiasis (PTT) at 6 weeks or 12 months by baseline TT severity**			
Overall*	347 (15.4)	504 (22.0)	298 (13.1)
Mild baseline TT	96 (9.5)	168 (15.1)	81 (7.7)
Moderate baseline TT	63 (14.9)	94 (24.5)	56 (12.7)
Severe baseline TT	187 (22.7)	242 (30.5)	161 (20.7)
**Moderate/severe eyelid contour abnormality (ECA) at final visit by baseline TT severity** †			
Overall	217 (9.6)	139 (6.1)	253 (11.2)
Mild baseline TT	80 (7.9)	56 (5.0)	103 (9.8)
Moderate baseline TT	48 (11.4)	17 (4.4)	56 (12.7)
Severe baseline TT	89 (10.8)	65 (8.2)	93 (12.0)
**Granuloma by 12 months**	147 (6.5)	118 (5.2)	170 (7.5)
**Characteristics of Eyes with PTT at First Visit with PTT [n(%)]**			
**First visit where PTT was detected**			
6-week visit	47 (13.5)	101 (20.0)	59 (19.8)
12–18-month visit	300 (86.5)	403 (80.0)	239 (80.2)
**PTT severity** ‡			
Mild	279 (80.4)	390 (77.4)	222 (74.5)
Moderate	36 (10.4)	63 (12.5)	43 (14.4)
Severe	32 (9.2)	50 (9.9)	33 (11.1)
**Number of eyelashes touching the globe**			
None	19 (5.5)	17 (3.4)	16 (5.4)
1–2	226 (65.1)	307 (60.9)	172 (57.7)
3–4	64 (18.4)	96 (19.0)	66 (22.1)
5–9	26 (7.5)	57 (11.3)	28 (9.4)
10+	12 (3.5)	27 (5.4)	16 (5.4)
**Evidence of epilation**			
None	307 (88.5)	457 (90.7)	257 (86.2)
<1/3 of eyelid	20 (5.8)	19 (3.8)	24 (8.1)
1/3-2/3 of eyelid	10 (2.9)	12 (2.4)	8 (2.7)
>2/3 of eyelid	10 (2.9)	15 (3.0)	9 (3.0)
**PTT location(s)** ‡			
Nasal	170 (49.0)	224 (44.4)	163 (54.7)
Central	183 (52.7)	308 (61.1)	128 (43.0)
Temporal	91 (26.2)	131 (26.0)	88 (29.5)

Abbreviations: BLTR: bilamellar tarsal rotation; TT: trachomatous trichiasis; PLTR: posterior lamellar tarsal rotation; PTT: Post-operative trachomatous trichiasis. Missing data: granuloma, 2 BLTR-5 eyes; PTT severity(6wk/12mo) and epilation, 1 BLTR-5 eye.

*1 BLTR-3 eyelid missing baseline TT severity is included in overall count but not in counts stratified by baseline TT severity.

†1 BLTR-5 eyelid and 1 PLTR eyelid missing baseline TT severity are included in overall moderate/severe ECA counts but not in moderate/severe ECA counts stratified by baseline TT severity.

‡Based on trichiatic eyelashes and epilation; 1 BLTR-5 excluded due to missing epilation data.

Unadjusted analyses and those adjusted for baseline TT severity and participant age yielded equivalent results. Overall, the difference in PTT incidence following BLTR-3 versus PLTR was not statistically different or clinically meaningful (adjusted risk difference [BLTR-3 minus PLTR] [aRD]: 1.8% (98.3%CI: -0.5–4.2%); adjusted OR [aOR] (98.3%CI: 1.20 (0.96–1.50); p = 0.058); however, the risk of PTT was significantly higher for BLTR-5 compared to both BLTR-3 (aRD: 6.7% (3.9–9.4%); aOR: 1.64 (1.34–2.01); p<0.0001) and PLTR (aRD: 8.6% (5.9–11.3%); aOR: 1.96 (1.58–2.43); p<0.0001) (**Table 3**).

**Table 3:** Unadjusted and Adjusted Model Results: Odds Ratios (98.3% Confidence Interval; p-value).

	Post-operative Trichiasis		Eyelid Contour Abnormality†		Pyogenic Granuloma‡	
Comparison	Unadjusted	Adjusted*	Unadjusted	Adjusted*	Unadjusted	Adjusted*
BLTR-3 vs. PLTR	1.21 (0.97–1.51; 0.040)	1.20 (0.96–1.50; 0.058)	0.84 (0.65–1.08; 0.094)	0.83 (0.64–1.07; 0.076)	0.86 (0.64–1.17; 0.24)	0.86 (0.64–1.17; 0.25)
BLTR-5 vs. PLTR	**1.91 (1.54–2.36; <0.0001)**	**1.96 (1.58–2.43; <0.0001)**	**0.50 (0.38–0.67; <0.0001)**	**0.50 (0.37–0.66; <0.0001)**	**0.68 (0.49–0.93; 0.0037)**	**0.68 (0.50–0.94; 0.0045)**
BLTR-5 vs. BLTR-3	**1.58 (1.29–1.93; <0.0001)**	**1.64 (1.34–2.01; <0.0001)**	**0.60 (0.45–0.80; <0.0001)**	**0.60 (0.45–0.81; <0.0001)**	0.78 (0.57–1.09; 0.08)	0.79 (0.57–1.10; 0.087)

Abbreviations: BLTR: Bilamellar tarsal rotation; PLTR: Posterior lamellar tarsal rotation.

*Adjusted for baseline trichiasis severity and age

†Moderate or severe ECA at final study visit (6 weeks or 12–18 months)

‡At 2 weeks, 6 weeks and/or 12–18 months follow up visit. If an eye developed a granuloma at two time points, it is only included once.

#### Eyelid contour abnormality

At the final study visit, 8.9% of eyelids (n = 609) had a moderate or severe ECA (**Table 2**). Unadjusted analyses and those adjusted for baseline TT severity and age showed BLTR-5 surgeries had significantly less risk of developing a moderate or severe ECA than either PLTR (RD: -4.9% (98.3%CI: -6.9 to -3.0%); aOR:0.48 (98.3%CI: 0.37–0.62); p<0.0001) or BLTR-3 (aRD: -3.3% (98.3%CI: -5.2 to -0.01%); aOR:0.63 (98.3%CI: 0.48–0.83); p<0.0001) (**Table 3**). Adjusted analyses stratified by surgeon type showed that among dually-trained surgeons, both BLTR-5 and BLTR-3 had a lower risk of ECA compared to PLTR, with BLTR-5 having the lowest risk. However, among conversion surgeons, there was no meaningful difference in ECA rates across the three procedures (**Table 4**).

**Table 4:** Post-operative Trachomatous Trichiasis (PTT) and Eyelid Contour Abnormality (ECA) Incidence by Surgeon Training Group.

	BLTR-3		BLTR-5		PLTR	
Total study eyes with follow up		2,257		2,290		2,268
	N	n(%)	N	n(%)	N	n(%)
**PTT incidence**						
Dually-trained (overall)	1,493	240 (16.1)	1,527	317 (20.8)	1,490	168 (11.3)
Surgeon 1	407	89 (21.9)	451	88 (19.5)	420	45 (10.7)
Surgeon 2	196	23 (11.7)	164	26 (15.9)	174	19 (10.9)
Surgeon 3	502	77 (15.3)	502	95 (18.9)	487	46 (9.5)
Surgeon 4	388	51 (13.1)	410	108 (26.3)	409	58 (14.2)
Conversion from BLTR to PLTR*^†^ (overall)	760	107 (14.0)	760	187 (24.5)	776	130 (16.7)
Surgeon 5	544	95 (17.5)	542	145 (26.8)	535	108 (20.2)
Surgeon 6	216	12 (5.6)	218	42 (19.3)	241	22 (9.1)
**Moderate/severe ECA incidence**						
Dually-trained (overall)	1,493	136 (9.1)	1,527	70 (4.6)	1,490	187 (12.6)
Surgeon 1	407	33 (8.1)	451	24 (5.3)	420	62 (14.8)
Surgeon 2	196	18 (9.2)	164	4 (2.4)	174	12 (6.9)
Surgeon 3	502	51 (10.2)	502	21 (4.2)	487	56 (11.5)
Surgeon 4	388	34 (8.8)	410	21 (5.1)	409	57 (13.9)
Conversion from BLTR to PLTR*^†^ (overall)	760	81 (106)	760	69 (9.0)	776	66 (8.5)
Surgeon 5	544	54 (9.9)	542	45 (8.3)	535	40 (7.5)
Surgeon 6	216	27 (12.5)	218	24 (11.0)	241	26 (10.8)

* Conversion surgeons first learned bilamellar tarsal rotation (BLTR) and later learned posterior lamellar tarsal rotation (PLTR). Dually-trained surgeons learned both procedures simultaneously.

† Excludes 4 BLTR-3, 3 BLTR-5, and 2 PLTR eyes operated by conversion surgeons who performed fewer than 10 surgeries.

#### Pyogenic granuloma

Over the study period, 6% of eyelids (n = 435) developed a pyogenic granuloma; 96% of granulomata developed between the 2- and 6-week visits. In unadjusted and adjusted analyses, BLTR-3 and PLTR had similar granuloma risk, while BLTR-5 had a significantly lower risk than PLTR (**Table 3**). Stratified analyses adjusted for baseline TT severity showed the same pattern among dually-trained surgeons, but no difference among the conversion surgeons.

#### Sensitivity and surgeon-type analyses

ECA analyses utilizing the field grade were nearly identical to the photograde-based analyses [adjusted OR (98.3% CI): BLTR 3 vs PLTR: 0.76 (0.60–0.96); BLTR-5 vs PLTR (0.48 (0.37–0.62), BLTR-5 vs BLTR 30.63 (0.48–0.83)]. Additionally, including the 21 individuals (27 eyes) assigned to id numbers out of order did not meaningfully change any study results. Per-protocol analyses including the 17 eyes operated with a non-assigned procedure also showed similar results.

Since surgeon training has been an area of interest, we also looked at whether differences in the order surgeons learned the procedure impacted their outcomes. Four dually-trained and three conversion surgeons participated in the trial; however, one conversion surgeon only performed nine surgeries and is excluded from these analyses. Among the six primary surgeons, post-hoc analyses stratified by surgeon type and adjusted for baseline TT severity showed that BLTR-5 had the highest incidence of PTT among the three procedures for both surgeon types (**Tables** 4 and 5). Overall, PTT rates for dually-trained surgeons were lower with PLTR than BLTR-3 (12.0% vs. 17.1%), while PTT rates for conversion surgeons who first learned BLTR were similar on BLTR-3 and PLTR (15.0% and 17.2%); **Tables** 4 and 5). However, PTT rates varied substantially across surgeons and within the dually-trained group, only two of the four surgeons performed notably better on PLTR compared with BLTR-3 when examining PTT rates.

**Table 5:** Secondary Analyses: Adjusted* Logistic Regression Models Stratified by Surgeon Training.

		#Events (% of surgeries)		Odds Ratios (98·3% Confidence Interval)	#Events (% of surgeries)		Odds Ratios (98·3% Confidence Interval)	#Events (% of surgeries)		Odds Ratios (98·3% Confidence Interval)
**Group 1 vs.** **Group 2**	**Surgeon Training Experience** †	**Group 1**	**Group 2**	**Post-operative Trichiasis**	**Group 1**	**Group 2**	**Eyelid Contour Abnormality**	**Group 1**	**Group 2**	**Pyogenic Granuloma**
BLTR-3 vs. PLTR	Dually-trained	240 (16.1)	168 (11.3)	1.48 (1.11–1.97; 0.001)	136 (9.1)	187 (12.6)	0.68 (0.50–0.93; 0.003)	104 (6.9)	138 (9.2)	0.74 (0.52–1.05; 0.040)
	Conversion	107 (14.0)	130 (16.7)	0.83 (0.58–1.21; 0.24)	81 (10.6)	66 (8.5)	1.23 (0.78–1.94; 0.27)	43 (5.6)	32 (4.1)	1.38 (0.75–2.52; 0.21)
BLTR-5 vs. PLTR	Dually-trained	317 (20.8)	168 (11.3)	2.14 (1.62–2.81; <0.0001)	70 (4.6)	187 (12.6)	0.32 (0.22–0.47; <0.0001)	83 (5.4)	138 (9.2)	0.57 (0.39–0.83; 0.0003)
	Conversion	187 (24.5)	130 (16.7)	1.73 (1.23–2.43; 0.0001)	69 (9.0)	66 (8.5)	1.06 (0.67–1.69; 0.73)	35 (4.5)	32 (4.1)	1.15 (0.61–2.17; 0.60)
BLTR-5 vs. BLTR-3	Dually-trained	317 (20.8)	240 (16.1)	1.44 (1.13–1.85; 0.0004)	70 (4.6)	136 (9.1)	0.47 (0.32–0.69; <0.0001)	83 (5.4	104 (6.9)	0.77 (0.52–1.14; 0.11)
	Conversion	187 (24.5)	107 (14.0)	2.08 (1.46–2.94; <0.0001)	69 (9.0)	81 (10.6)	0.87 (0.55–1.37; 0.45)	35 (4.5)	43 (5.6)	0.84 (0.47–1.50; 0.46)

*Adjusted for baseline trichiasis severity and age.

†Conversion surgeons first learned bilamellar tarsal rotation (BLTR) and later learned posterior lamellar tarsal rotation (PLTR). Dually-trained surgeons learned both procedures simultaneously.

### Adverse events

During one PLTR surgery, the surgeon accidentally cut the eyelid margin. The margin was repaired by placing a single 4–0 silk suture that was removed at the 2-week post-operative visit. We last saw this participant at six weeks because they died before the 12-month visit, and the eyelid had a moderate ECA at that time. Four eyelids in the BLTR-3 arm and eight in the BLTR-5 arm had excessive bleeding, defined as soaking more than two gauze pads after tying the sutures and applying the gauze eye patches. We treated these eyes with additional pressure applied to the wound, and all resolved without further complication. Infection, characterized by the presence of pus, was uncommon, with 1.2% in BLTR-3, 1.3% in BLTR-5 and 0.7% in PTLR reported two weeks after surgery. One individual was hospitalized, and 69 individuals died. Of these 69 individuals that died, one also had a prior hospitalization. All deaths and hospitalizations were deemed not related to the study procedure.

## Discussion

In this large, randomized clinical trial, overall, we found no significant difference in PTT risk within one year after surgery comparing standard BLTR surgery (BLTR-3) versus PLTR. However, one-year PTT risk was significantly higher following BLTR surgery when the incision height was raised to 5 mm (BLTR-5) compared to both BLTR-3 and PLTR. The higher PTT incidence with BLTR-5 was unexpected, as previous research by our team suggested that PTT incidence may be significantly lower when the incision scar height is ≥4.5 mm [3]. However, the prior data were based on incision scar height measured a year after surgery, not the incision height at the time of surgery. It is possible that incision height and one-year scar height are not completely correlated due to eyelid remodeling after surgery. In the current trial, we measured scar heights 12 months after surgery, and in the future, we plan to conduct an analysis evaluating the association between measured incision height and scar height.

Prior to this trial, limited data were available on the impact of incision height on surgical outcomes. While the WHO *Trichiasis Surgery for Trachoma* manual [2] indicates that the incision should be made 3 mm above the eyelid margin, we previously observed wide variation in the way that surgeons make their incision [9]. Many surgeons use the width of the scalpel blade as a guide for where to make the incision, which often results in an incision at about 4.5mm—50% higher than the recommended 3 mm target. Within the current trial, we strictly monitored BLTR incision height by requiring surgeons to mark the external incision height with a surgical pen and calipers. After incision, the surgeon then measured the internal incision height with a ruler and documented the measured height with photographs that masked graders reviewed as well. Because the pen mark can vary in thickness, and the preciseness of the cut can cause variation, the surgeon then measured the internal incision height with a ruler and documented the measured height with photographs that masked graders reviewed as well.

Our findings differ from a prior randomized trial that compared PLTR with standard BLTR surgery. In that trial, PLTR was associated with a significantly lower one-year risk of PTT than standard BLTR surgery (13% vs 22%) [4], while overall our study found no meaningful difference in one-year PTT risk between PLTR and BLTR-3 (13% vs. 15%). Of note, the PTT risk following PLTR is the same between the two studies, while our BLTR-3 PTT rate (15%) is substantially lower than that of the prior trial (22%). Both studies were conducted in Ethiopia in populations with similar baseline TT severity. One possible explanation for the difference in our findings is the surgeon population employed for the two trials. In the current trial, surgeons were either first taught BLTR and then learned PLTR, or they learned both procedures simultaneously. In the previous trial, all surgeons learned PLTR first and later learned BLTR. In both trials, these “conversion” surgeons performed the new procedure approximately 100 times prior to conducting trial-related surgeries.

Another explanation is the difference between the Waddell clamp and the TT clamp. The previous trial used the Waddell clamp, and our trial used the TT clamp. With the Waddell clamp, the eyelid margin rests upon a bar that is perpendicular to the plate. This design difference may have impacted the angle of the blade and the resultant tarsal incision height when the Waddell clamp was used. The vertical bar is an obstacle that encourages the surgeon to angle the blade perpendicular to the plate or away from the bar. When the blade is angled away from the bar, the sharp edge of blade is directed downward, resulting in a lower tarsal incision. Since the previous trial measured the incision height from the skin side only, the actual incision height in the tarsus could have been different (likely shorter) and ultimately may have modified the outcome. This shorter internal incision height may help explain their ancillary finding that higher incision heights were associated with a lower risk of PTT for PLTR, but a higher risk for BLTR surgeries, given that the measurements are based on the external incision height. Head-to-head testing of the Waddell clamp and the TT clamp would be needed to determine whether the design differences are important.

In our study, stratified analyses suggest that when surgeons learned both procedures simultaneously, the risk of PTT is generally lower following PLTR than BLTR, while those who first learned BLTR tended to perform similarly on BLTR-3 and PLTR or slightly better on BLTR-3. Our findings suggest that surgeons tend to perform better on BLTR when they learn that procedure first. Interestingly, in both our study and Habtamu et al [4], the incidence of PTT by surgeon was fairly consistent over time, despite each surgeon performing hundreds of surgeries. This is similar to a recent study of radical prostatectomy that found case volume of experienced surgeons did not necessarily correlate with positive outcomes [10]. In both TT trials, the number of surgeons is relatively low, making it difficult to make conclusive decisions regarding the impact of the training order.

This study also investigated the risk of ECAs and pyogenic granuloma formation. We saw no differences in risk of moderate to severe ECA or granuloma comparing BLTR-3 versus PLTR. However, both were less common following BLTR-5 as compared to either BLTR-3 or PLTR. ECAs were most common in the PLTR group and least common in the BLTR-5 group (11% vs 6%). This finding also differs from the Habtamu study described above. That study found a higher overall rate of ECA in PLTR (24% vs 18%), but among “clinically-significant” ECAs, they did not see a meaningful difference (6% vs. 8%). The absolute ECA rates in the two studies cannot be directly compared because the two trials utilized different approaches to evaluating ECAs; however, the relative difference between two procedures in the same trial should be comparable. ECAs and PTT typically do not present together since they are often associated with opposing forces. ECAs are more common when the eyelid is over-corrected, while PTT is more common when the eyelid is under-corrected. Prior to this trial, some experts expressed concern that raising the BLTR incision height would increase risk of ECA because the distal tarsal fragment would be larger, and the ECA could be magnified by the larger fragment size. However, our results demonstrated the opposite. In contrast to pre-trial thoughts, the lower ECA risk in the BLTR-5 group could be explained by the larger fragment being more difficult to distort into an ECA than the thinner, more bendable fragment created by a lower incision. Across procedures, ECA rates increased with increasing pre-operative TT severity. We anticipated this finding, since eyelids with more severe TT typically have more tarsal conjunctival scarring [12], making the surgery more challenging. Furthermore, in groups where we saw higher PTT rates, ECA rates were lower. This finding also is expected, given that ECA is more likely to develop when the eyelid is over-corrected, while PTT is more common when the eyelid is under-corrected [12]. The overall rate of granuloma formation in this study (6%) was similar to the rates in two previous studies conducted in Ethiopia [4,11]. In the current study, only BLTR-5 had a significantly lower rate of granuloma than PLTR, and this was only seen in the dually-trained surgeon group.

This study has several strengths. MTSS is the largest clinical trial to compare the two most-commonly used trichiasis surgery procedures, BLTR and PLTR. We employed strict procedure monitoring, including photographing all study outcomes to ensure accuracy of outcomes reporting. All surgeons were certified by study ophthalmologists (FA, AS, SLM) prior to performing trial-related surgeries and our follow up rates were high. This study also has some limitations. Our analyses stratified by training type are limited to four dually-trained surgeons and two conversion surgeons who performed most of the surgeries in this group. While we had originally trained four conversion surgeons, two were not able to participate extensively in the trial given other responsibilities and the need to travel throughout SNNPR during a two-year recruitment period. This limits our ability to interpret findings based on surgeon type. More research is needed on the impact of the order in which procedures are taught on the outcomes for each of those procedures.

## Conclusions

We do not recommend increasing the BLTR incision height from 3 mm to 5 mm, given that it has the highest PTT rate across the three groups. In our setting, where four of the six surgeons trained on both procedures simultaneously, we do not see meaningful differences in PTT rates between BLTR-3 and PLTR overall. The small number of surgeons in this study limits our ability to adequately address the impact of training order on study outcomes. More evidence is needed to assess the impact of training order on surgical performance.

## Supporting information

S1 CONSORT Checklist Checklist of information to include when reporting a randomized trial. (PDF)
